# Transcriptome Analysis of Porcine Immune Cells Stimulated by Porcine Reproductive and Respiratory Syndrome Virus (PRRSV) and *Caesalpinia sappan* Extract

**DOI:** 10.3390/ijms252212285

**Published:** 2024-11-15

**Authors:** Chaiwat Arjin, Patipan Hnokaew, Patchara Tasuksai, Marninphan Thongkham, Kidsadagon Pringproa, Jirapat Arunorat, Terdsak Yano, Mintra Seel-audom, Pornchai Rachtanapun, Korawan Sringarm, Phongsakorn Chuammitri

**Affiliations:** 1 Department of Animal and Aquatic Sciences, Faculty of Agriculture, Chiang Mai University, Chiang Mai 50200, Thailand; chaiwat.arjin@cmu.ac.th (C.A.); patipan.hnokaew@cmu.ac.th (P.H.); patchara_ta@cmu.ac.th (P.T.); marninphan_t@cmu.ac.th (M.T.); mintra.s@cmu.ac.th (M.S.-a.); 2 Office of Research Administration, Chiang Mai University, Chiang Mai 50200, Thailand; 3 Veterinary Academic Office, Faculty of Veterinary Medicine, Chiang Mai University, Muang, Chiang Mai 50100, Thailand; kidsadagon.p@cmu.ac.th (K.P.); jirapat.a@cmu.ac.th (J.A.); terdsak.yano@cmu.ac.th (T.Y.); 4 School of Agro-Industry, Faculty of Agro-Industry, Chiang Mai University, Chiang Mai 50100, Thailand; pornchai.r@cmu.ac.th; 5 Center of Excellence in Agro Bio-Circular-Green Industry (Agro BCG), Agro-Industry, Chiang Mai University, Chiang Mai 50100, Thailand

**Keywords:** transcriptome, PRRS virus, porcine PBMC, *Caesalpinia sappan*, differentially expressed genes

## Abstract

The current level of knowledge on transcriptome responses triggered by *Caesalpinia sappan* (CS) extract in porcine peripheral blood mononuclear cells (PBMCs) after porcine reproductive and respiratory syndrome virus (PRRSV) infection is limited. Therefore, in the present study, we aimed to detect significant genes and pathways involved in CS extract supplementation responsiveness of PBMCs after PRRSV infection. RNA sequencing was conducted on PBMCs, which were isolated from six weaned piglets. The resultant transcriptional responses were examined by mRNA sequencing. Differential expression analysis identified 263 and 274 differentially expressed genes (DEGs) between the PRRSV and CTRL groups, and the PRRSV+CS and CTRL groups, respectively. Among these, *ZNF646* and *KAT5* emerged as the most promising candidate genes, potentially influencing the interaction between PRRSV-infected PBMCs and CS extract supplementation through the regulation of gene networks and cellular homeostasis during stress. Two pathways were detected to be associated with CS extract supplementation responsiveness: the cellular response to stress pathway and the NF-kB signaling pathway. Consequently, our study reveals a novel mechanism underlying cellular stress response and the NF-κB signaling pathway in PRRSV-infected PBMCs, and identifies a potential application of CS extract for activating the NF-κB signaling pathway. In conclusion, by supplementing CS extract in PBMC cells infected with PRRSV, we found that CS extract modulates PRRSV infection by inducing cellular stress, which is regulated by the NF-κB signaling pathway. This induced stress creates an adverse environment for PRRSV survival. This study contributes to a deeper understanding of the therapeutic targets and pathogenesis of PRRSV infection. Importantly, our results demonstrate that CS extract has the potential to be a candidate for modulating PRRSV infection.

## 1. Introduction

Porcine reproductive and respiratory syndrome virus (PRRSV) is an enveloped single-stranded positive-sense RNA virus that belongs to the *Arteriviridae* (order *Nidovirales*) family [1]. PRRSV is a major pathogen that causes porcine reproductive and respiratory syndrome (PRRS), a condition that affects pig production and causes economic losses worldwide. PRRSV primarily causes reproductive failure in sows and respiratory dysfunction in pigs at various stages, as well as predisposes pigs to bacterial and other viral infections [2,3]. PRRSV shows a very narrow host cell tropism, infecting only specific porcine macrophage subsets [4]. At present, there is no medication available to manage this disease. Vaccination is currently the most effective strategy for preventing PRRSV. However, the rapid evolution of PRRSV strains, coupled with slow vaccine development and frequent strain recombination [5], limits the effectiveness of current vaccines in providing complete protection against PRRSV infection [6]. The continuous emergence of new strains, low cross-protection between strains, and the potential for attenuated vaccines to regain virulence further complicate PRRSV control [7]. The emergence of these elusive PRRSV strains significantly impedes PRRSV prevention and control, complicating vaccine selection and vaccination strategy development in the pig industry. The overuse of antibiotics in pig production poses significant risks to the environment, food safety, and public health [8]. Thus, there is a critical need to identify non-antibiotic natural compounds to effectively prevent and treat PRRS, offering a novel approach to PRRSV control in clinical settings. Natural products have been utilized to control PRRSV in both in vitro and in vivo studies [2,3,9,10,11,12].

According to our previous research, *Caesalpinia sappan* (CS) extract has the potential to inhibit PRRSV in vitro by preventing PRRSV replication in the MARC-145 cell line [9]. Additionally, the semi-purification of CS demonstrated potential antiviral activity and inhibited PRRSV replication in a MARC-145 monolayer at 72 h post-infection. This fraction was subsequently identified as an active compound, including byakangelicin, brazilin, and naringenin [13]. In particular, brazilin, the most significant bioactive compound of CS, demonstrated the ability to obstruct the CD163^ΔSRCR5^ receptor, thereby preventing the infection of cells by PRRSV [14]. CS is a plant that is a member of the *Leguminosae* family. This medicinal plant is widely recognized and is propagated in tropical Asian nations, such as China, India, Myanmar, and Thailand. The dried heartwood of CS has been employed in oriental remedies, such as ayurveda and traditional Chinese medicine, for centuries [15]. Moreover, CS has been evaluated in piglets infected with PRRSV, and it was found to enhance the piglet’s immunity by increasing the titer of antibodies and lymphocytes against PRRSV [15].

The transcriptomic analysis is one of the molecular techniques used to study the structure, function, and evaluation of the transcriptome. Transcriptome analysis of PBMCs reveals not only the primary immune response of leukocytes, but also the extent and dynamics of antigen-induced gene expression changes [16]. The improved comprehension of host defense and disease resistance is facilitated by the identification of the key genes and signaling and metabolic pathways that are affected [17]. Nevertheless, there is no information regarding the mechanism by which bioactive compound molecules interact with CS to improve the immunity of pigs. The molecular responses of porcine immune cells to viral infection and potential therapeutic interventions are the subject of a critical investigation through the transcriptome analysis of PRRSV-stimulated PBMCs in conjunction with CS extract. The objective of this investigation is to clarify the complex mechanisms that underlie the interaction between the host immune response and viral infection, thereby informing the development of new immunomodulatory strategies and treatment options for PRRSV.

## 2. Results

### 2.1. Detection and Characterization of Differentially Expressed Genes (DEGs)

**Figure 1:**
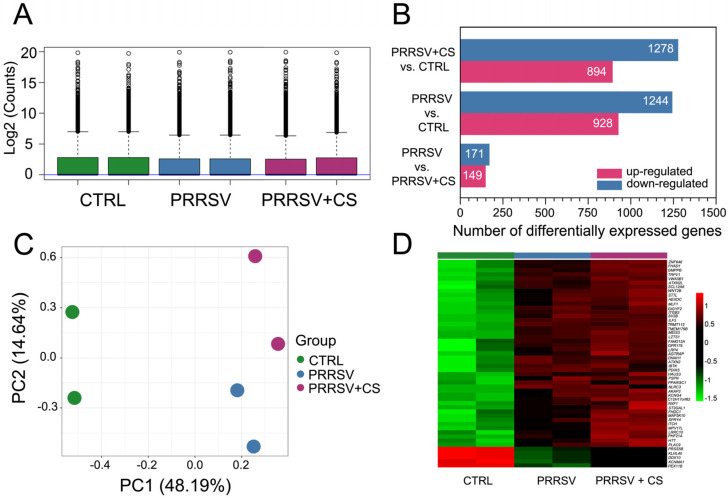
Characterization of differential expression analysis. (**A**) Raw read counts (Log2) of three treatment groups generated by treatment group enrichment analysis. (**B**) Bar diagram illustrates representative up- and downregulation for pairwise comparison between three treatment groups, absolute LFC of genes > 2 from DESeq2. (**C**) Principal component analysis (PCA) of gene expression profile of all samples. Control PBMS cells (CTRL) are shown in green circles; PRRSV-infected cells (PRTE) are presented in blue circles; PRRSV-infected cells with CS extract supplementation (PRTE+CS) are illustrated in purple circles. (**D**) Heatmap constructed using LFC of a total of 50 genes in matched comparisons; colors indicate the up- (red) and downregulation (green) of gene expression.

The expression levels were quantified by measuring transcript abundance in the control group of PBMC cells (CTRL; GSM8529034, GSM8529035), the PRRSV-infected group (PRRSV; GSM8529036, GSM8529037), and the group of PRRSV-infected cells with CS extract supplementation (PRRSV+CS; GSM8529038, GSM8529039). After filtering, a total of 16,926 genes were retained from the initial 29,886 gene entries for the differential expression analysis. The raw read counts (Log2) indicate the PRRSV group and PRRSV+CS group were not different from the CTRL group. (Figure 1A). The three treatment comparisons (PRRSV+CS VS CTRL, PRRSV VS CTRL, and PRRSV+CS VS PRRSV) yielded 2172 DEGs (894 up- and 1278 downregulated), 2172 DEGs (928 up- and 1244 downregulated), and 320 DEGs (149 up- and 171 downregulated), respectively (Figure 1B). Principal component analysis (PCA) is a dimensionality reduction technique used to condense a large set of variables into a smaller set while preserving most of the original information. The PCA plot, utilizing the first and second principal components, clearly illustrated the distinction between PRRSV+CS and PRRSV groups compared with the CTRL group (Figure 1C). In addition, a heatmap diagram based on 50 genes with the highest log_2_ fold change (LFC) of DEGs revealed approximately inverse directions of PRRSV+CS and PRRSV effects (Figure 1D).

### 2.2. Differentially Expressed Genes

**Figure 2:**
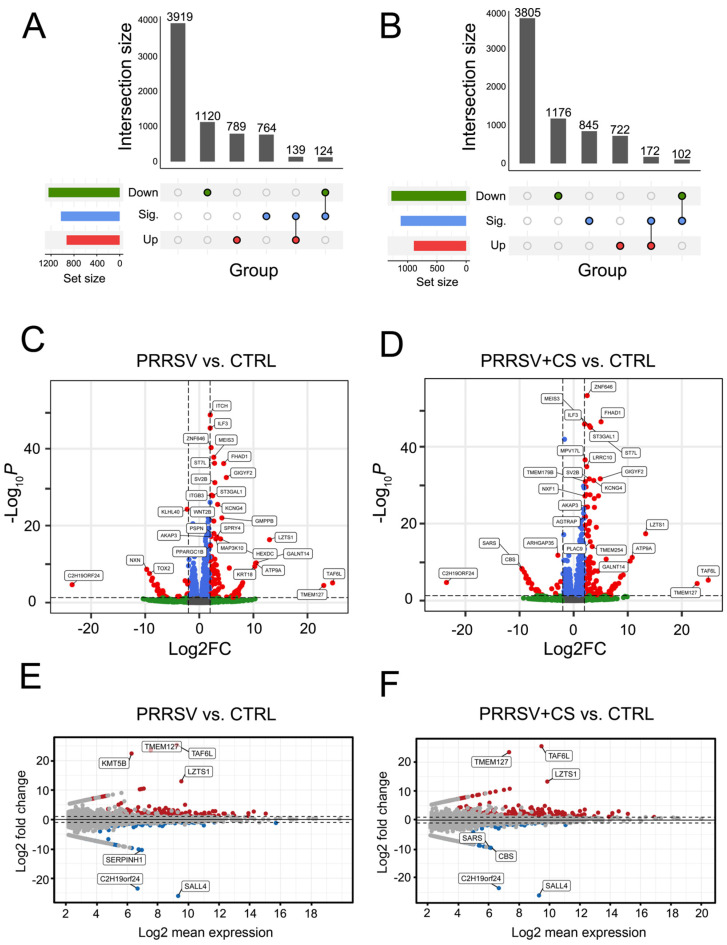
Summary plots for differential expression analysis using DESeq2. (**A**,**B**) UpSet plot illustrates unique and shared DEGs of pairwise comparisons PRRSV vs. CTRL (**A**), PRRSV+CS vs. CTRL (**B**). (**C**,**D**) Volcano plot shows statistically significant DEGs of pairwise comparisons PRRSV vs. CTRL (**C**), PRRSV+CS vs. CTRL (**D**). Red dots indicate significant up- and downregulation, absolute LFC > 2, FDR < 0.05. (**E**,**F**) MA plot from DESeq2 of pairwise comparisons PRRSV vs. CTRL (**E**), PRRSV+CS vs. CTRL (**F**), shows LFC (*y*-axis) and the Log_2_ mean expression (*x*-axis) across all samples. Absolute LFC of genes > 2 from DESeq2 is color highlighted. Red represents upregulation, blue represents downregulated genes, and grey dots represent no change.

Under the criteria of LFC > 2 and FDR < 0.05, 263 DEGs, including 139 upregulated and 124 downregulated DEGs, were identified as statistically significant by comparing the PRRSV and CTRL groups. (Figure 2A; Table S1). During the comparison of PRRSV+CS versus CTRL groups, 274 DEGs (172 up- and 102 downregulated) exhibited statistically significant up- or downregulation (Figure 2B; Table S2). The overall distribution of DEGs between PRRSV and PRRSV+CS compared with the CTRL group was inferred using a volcano plot (Figure 2C,D). Genes that exhibited an LFC greater than two in either direction and an FDR less than 0.05 were considered to be significantly differentially expressed. *ITCH*, a ubiquitination process, showed the most potent responsiveness to the PRRSV group, whereas *ZNF646*, implicated in the DNA-binding transcription factor activity, showed the most potent responsiveness to the PRRSV+CS group. The mean average (MA) plot (Figure 2E,F) showed that PRRSV-infected cells (PRRSV) and PRRSV-infected cells stimulated with CS extract (PRRSV+CS) directed a massive transcriptomic response. A cursory look at the top genes determined by the LFC > 10 showed the genes *TAF6L*, *TMEM127*, and *LZTS1* were upregulated in the PRRSV+CS group compared with the CTRL group. Similarly, these genes were upregulated in the PRRSV group compared to the CTRL group, with the *KMT5B* gene showing notably higher upregulation in this comparison. However, in the PRRSV and PRRSV+CS groups, the expression of the *C2H19orf24* and *SALL4* genes was significantly downregulated compared to the CTRL group. This result demonstrated that there were different gene expression patterns between the PRRSV group and PRRSV+CS compared with the CTRL group.

### 2.3. Gene Ontology (GO) Terms and Pathways Enriched by DEGs

**Figure 3:**
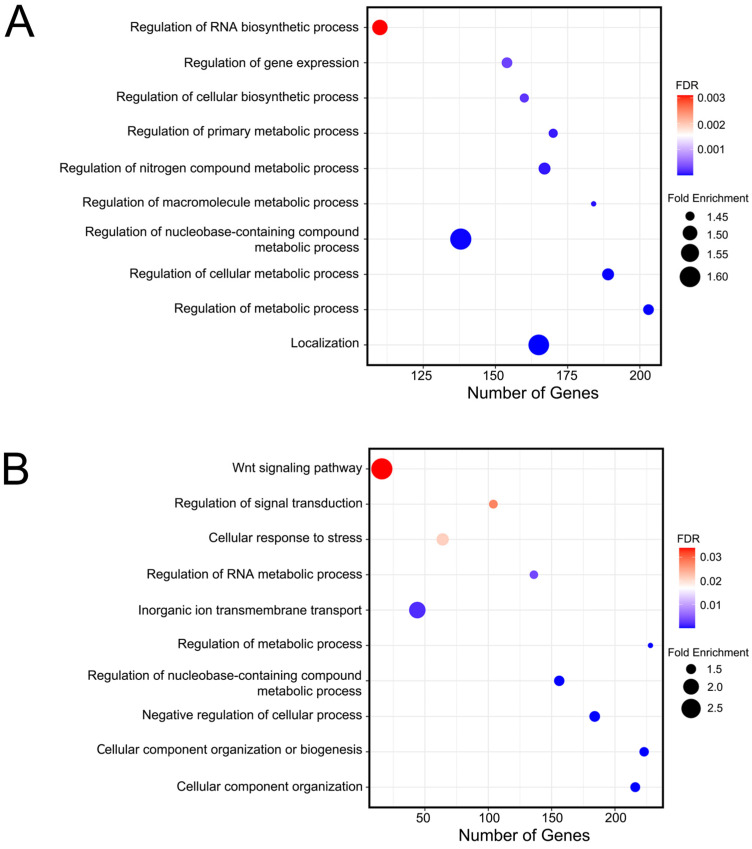
RNA-seq data reveal GO biological processes among differentially expressed genes between the PRRSV group and the CTRL group (**A**), and between the PRRSV+CS group and the CTRL group (**B**). The bubble plot represents the number of genes (*x*-axis), fold enrichment (bubble size), and FDR value (gradient colors) in each GO biological process term (*y*-axis). Representative bubbles were enriched at FDR < 0.05.

The GO analysis revealed that PRRSV-infected differentially expressed genes (upregulated) are involved in the process of active biological processes, such as localization (GO:0051179), regulation of nucleobase-containing compound metabolic process (GO:0019219), and regulation of RNA biosynthetic process (GO:2001141) (Figure 3A). During the comparison of PRRSV+CS versus CTRL groups, upregulated DEGs observed in PRRSV-infected cells with CS extract supplementation were involved with the enrichment of GO terms such as the Wnt signaling pathway (GO:0016055), inorganic ion transmembrane transport (GO:0098660), and cellular response to stress (GO:0033554) (Figure 3B). Overall, there were significantly altered transcripts participating in biological activation and differentiation, gene expression regulation, RNA biosynthesis, membrane transport, stress response, and cellular metabolic processes.

### 2.4. Global Gene Networks Analysis

**Figure 4:**
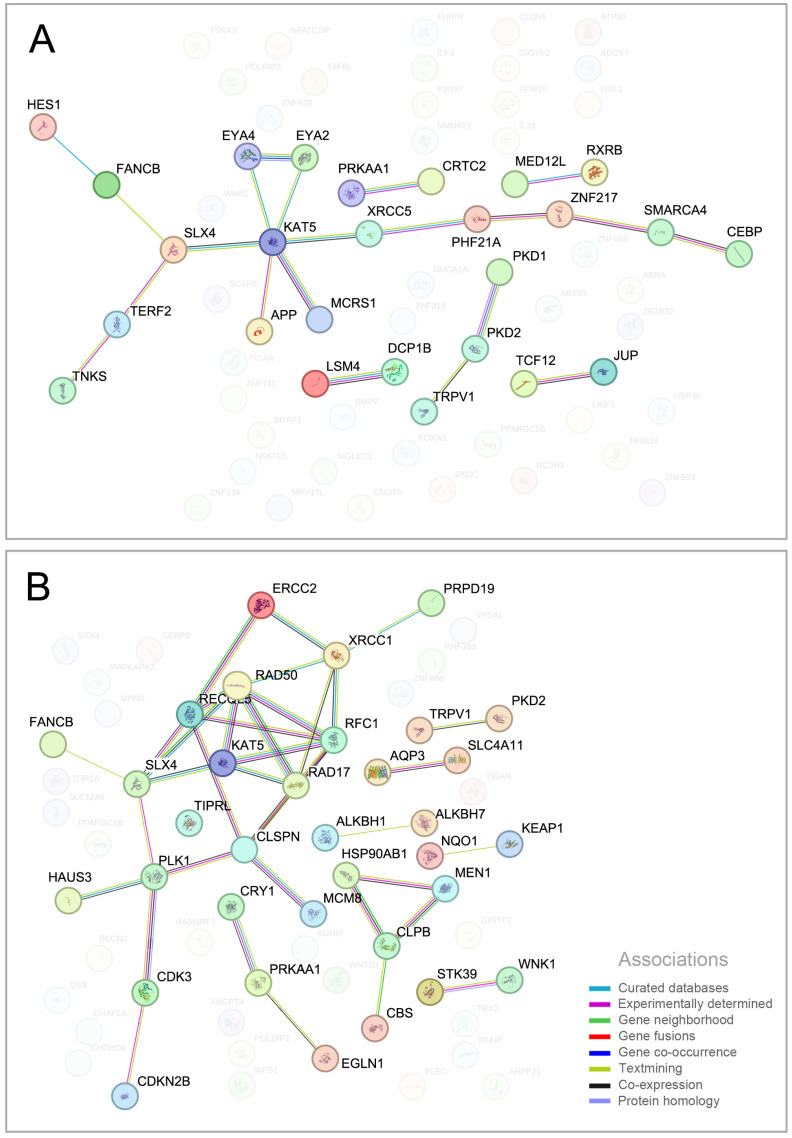
Protein–protein interaction network (PPI) of DEGs produced by STRING analysis. STRING analysis was used to analyze DEGs in PBMC cells of PRRSV versus CTRL (**A**) and PRRSV+CS versus CTRL (**B**). Differently colored lines represent eight types of evidence used in predicting associations. Blue line: curated databases evidence; dark purple: experimentally determined evidence; green line: gene neighborhood evidence; red line: gene fusion evidence; dark-blue line: gene co-occurrence evidence; light-green line: textmining evidence; black line: co-expression evidence; and purple line: protein homology evidence.

The simplified network of PRRSV-infected cells and PRRSV-infected cells with CS extract supplementation in PBMCs is presented in Figure 4. Protein–protein interaction (PPI) analysis was performed, and we observed significant interplay in a subset of 15 proteins encoded by upregulated DEGs between the PRRSV group and CTRL group (Figure 4A). In the network, the proteins encoded by HES1, FANCB, SLX4, TERF2, TNKS, KAT5, EYA4, EYA2, APP, MCRS1, XRCC5, PHF21A, ZNF217, SMARCA4, and CEBP were highly interconnected. Functional annotation revealed that hub genes in this module were implicated in cellular responses to stress. Network topology analysis of upregulated DEGs in PRRSV+CS vs. CTRL identified 16 highly interconnected hub proteins [Figure 4B], including CDKN2B, CDK3, HAUS3, PLK1, FANCB, SLX5, KAT5, CLSPN, MCM8, RAD17, RFC1, RECQL5, RAD50, ERCC2, XRCC1, and PRPF19. These proteins are functionally linked to telomere maintenance, DNA metabolism, and nucleobase metabolism.

### 2.5. Transcript Profiles and RT-qPCR Validation of Selected Genes

**Figure 5:**
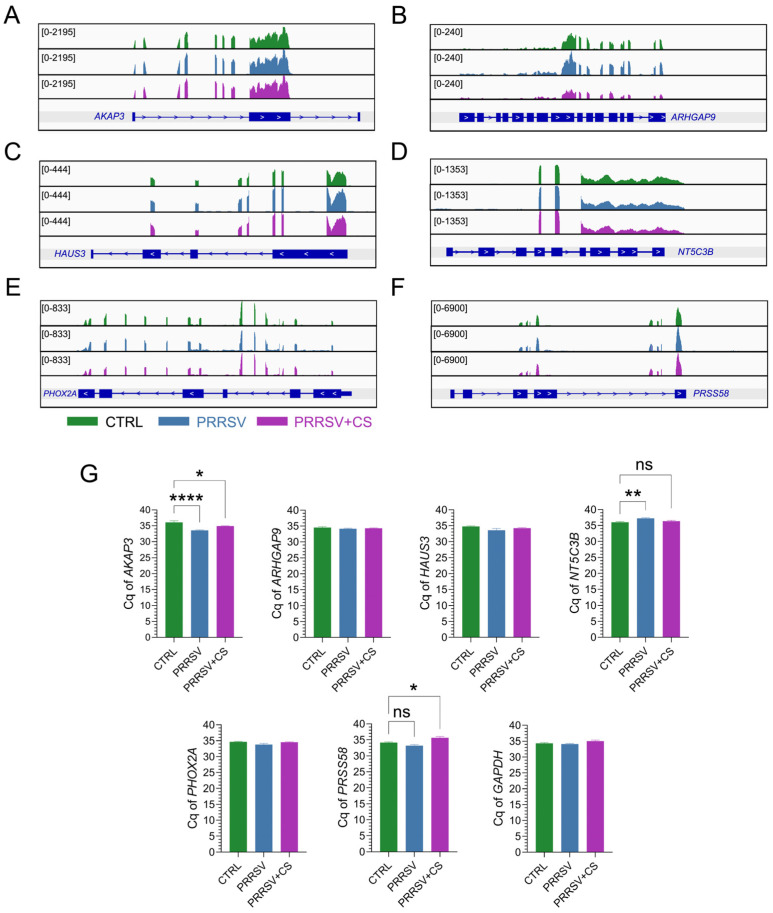
Gene landscapes of selected genomic regions and DEGs in PBMC cells infected with PRRSV (PRRSV) and PBMC cells infected with PRRSV with CS extract supplementation (PRRSV+CS) versus control samples (CTRL). Gene track views of raw mapped reads of *AKAP3* (**A**), *ARHGAP9* (**B**), *HAUS3* (**C**), *NT5C3B* (**D**), *PHOX2A* (**E**), and *PRSS58* genes (**F**). RNA-seq peaks of mRNA expression (read density) are shown and compared across six samples by genome browser (IGV) screenshots. Genomic coordinates of each gene are given underneath each gene track. (**G**) Bar graphs show *AKAP3*, *ARHGAP9*, *HAUS3*, *NT5C3B*, *PHOX2A*, and *PRSS58* transcript mRNA cycle quantification (Cq) by RT-PCR. Data are mean ± standard error mean (SEM). * *p* < 0.05, ** *p* < 0.01, **** *p* <0.001, ns = no significance.

To validate the expression level of genes estimated by RNAseq data, six genes, namely, *AKAP3*, *ARHGAP9*, *HAUS3*, *NT5C3B*, *PHOX2A*, and *PRSS58*, were selected for RT-qPCR confirmation to verify the gene expression level based on raw read counts of each treatment group. We utilized IGV to explore our comprehensive pig genome datasets and identify differential expression at the exonic transcript level. Exonic landscapes of the aforementioned genes were retrieved, incorporating genomic annotations and read counts (Figure 5A–F). For real-time qPCR analysis (Figure 5G), the expressions of the *AKAP3* mRNA of PRRSV and PRRSV+CS groups were significantly enhanced in comparison with the CTRL group. Meanwhile, the expression of *NT5C3B* and *PRSS58* mRNA was significantly decreased in the PRRSV and PRRSV+CS groups compared to the CTRL group, respectively. mRNA expression of *ARHGAP9*, *HAUS3*, and *PHOX2A* genes did not indicate any significance among treatment groups.

## 3. Discussion

PRRSV is a significant economic burden on the swine industry, causing reproductive failure and respiratory disease [18]. While vaccination is a common control strategy, it presents challenges such as the risk of vaccine-induced virulence and reliance on the host’s immune response [19,20]. The utilization of a natural product like the CS extract is an interesting choice because it has the potential to inhibit PRRSV in vitro by preventing PRRSV replication in the MARC-145 cell line, as demonstrated in our previous research [14]. For this reason, this study investigated genome-wide transcriptional changes in PBMCs associated with CS extract supplementation responses after PRRSV infection. To the best of our knowledge, this is the first study that comprehensively describes the CS extract influence of transcriptome responses to PRRSV infection in PBMCs. Finally, our results revealed several significant genes and biological processes involved in the CS extract responsiveness of PBMCs infected with PRRSV via next-generation sequencing technology.

The current study yielded a transcriptome dataset comprising 274 DEGs involved in PRRSV infection with CS extract supplementation. The reliability of this dataset was validated by RT-qPCR analysis of six selected genes (Figure 5G). The proportion of upregulated genes was higher than that of the downregulated genes. While the exact mechanism underlying this global upregulation remains unclear, we hypothesize that host genetics may play a role. Age and breed, for instance, are known to significantly influence the development of anti-PRRSV immunity [21,22]. Additionally, we hypothesize that the bioactive compounds of CS extract may influence the DEGs of PBMC infection. The exact mechanisms by which dietary bioactive compounds or their metabolites influence molecular targets remain unclear. While many of these changes occur at the gene expression level, it is hypothesized that their effects may be mediated by direct molecular interactions (signaling pathways or transcription factors) or, more likely, through epigenetic mechanisms (DNA methylation, histone modifications, microRNAs, or lncRNAs) [23,24,25].

GO enrichment analysis provides insights into the complex interplay of genes involved in host responses to various animal viruses [26,27,28,29]. In this study, GO analysis revealed several significant biological processes associated with PRRSV induction and CS extract stimulation. In the current study, our results showed that more than 10 biological processes were involved in PBMC-infected cells. Most DEGs were significantly enriched under the GO biological process of localization, nucleobase-containing compound metabolic process, and cellular biosynthetic process. Besides the previously mentioned GOs, we also found other GO terms, which were connected to gene expression and cellular metabolic pathways in PRRSV infection, in accordance with previous reports [30]. Interestingly, the GO terms of PBMC-infected cells stimulated with CS extract showed a marked increase in activity within the GO biological processes related to the cellular response to stress, regulation of signal transduction, and the Wnt signaling pathway. In particular, cellular response to stress plays a significant role in innate and adaptive immune responses [31], which was moderately upregulated in the PRRSV+CS group compared with the CTRL group. Cellular stress responses, activated by various stressors, include the oxidative stress response, heat shock response, unfolded protein response, and DNA damage response [32]. Proteins involved in these responses, such as those in the heat shock, ER stress, and DNA damage responses, interact with and regulate signaling pathways that activate both innate and adaptive immune responses. The effect of such regulation by cell stress proteins may dictate the inflammatory profile of the immune response during infection and disease [31]. Following RNA virus infection, viral RNA is recognized by Toll-like receptors (TLRs) or RIG-I-like receptors (RLRs), triggering antiviral innate immune responses [33]. Pathogen recognition receptors (PRRs) recognize viral RNA and activate protein kinase (TAK1) and binding proteins (TABs), leading to the activation of IKKα and IKKβ [34]. Among these kinases, IKKβ plays a crucial role in the classical NF-κB pathway, regulating NF-κB signaling and interferon production by phosphorylating IκB proteins. This phosphorylation event triggers the transcription of antiviral factors [35]. The canonical NF-κB pathway is essential for innate immune responses. NF-κB dimers, composed of p50, p52, p65, c-rel, and relB [36], are normally inactive in the cytoplasm due to interactions with inhibitory IκB proteins [37,38]. The classical NF-κB pathway, primarily regulated by IKKβ, involves the phosphorylation and subsequent degradation of IκB proteins [37]. This allows NF-κB dimers to translocate into the nucleus and activate the transcription of type I interferon and inflammatory cytokines [39].

To prolong its survival and replication, PRRSV has evolved strategies to evade the host’s immune response. The ongoing interaction between virus and host has shaped the viruses’ survival strategies [40,41]. To replicate and spread, the virus has developed various immune evasion mechanisms, disrupting cellular immune signaling pathways [42,43]. To date, numerous PRRSV nonstructural proteins have been found to be involved in the negative regulation of NF-κB activity, including Nsp1a, Nsp1β, Nsp2, Nsp4, and Nsp11 [42,44,45,46]. Recent research found that PRRSV Nsp4 inhibits the NF-κB signaling pathway by cleaving IKKβ, revealing a new mechanism by which PRRSV escapes host innate immunity in favor of its own survival; as a result, PRRSV-induced NF-κB activation can be interfered with and IFN-I can also be significantly inhibited [40]. It has been reported that activation of NF-κB is involved in the replication processes of a variety of viruses, such as dengue virus [47], classical swine fever virus [48], and African swine fever virus. While direct or indirect inhibition of NF-κB can suppress PRRSV replication [49], PRRSV-induced upregulation of TRME2 can inhibit NF-κB activity, leading to increased membrane CD163 expression and enhanced viral replication. These findings suggest that NF-κB activity may have diverse roles during PRRSV infection, and PRRSV may have evolved multiple strategies to manipulate NF-κB signaling for its own replication. This may explain the persistent nature of PRRSV infections in clinical settings [40]. In this study, we hypothesize that natural compounds in CS extract promote the structural integrity of IKKβ, leading to the activation of NF-κB activity and creating an environment adverse to PRRSV survival. This results in heightened activation of the cellular stress response in PBMC cells because the NF-κB pathway plays important roles in the control of stress response, inflammation, differentiation, apoptosis, cell growth, and many other physiological processes by regulating cellular signaling [50,51], which has guided the development and application of natural antiviral compounds. However, we will study the mechanism between natural compounds in CS extract and the activation of NF-kB expression in future research. For PPI analysis, our findings support the hypothesis regarding the ability of CS extract to activate the NF-kB signaling pathway. The *KAT5* gene encodes an essential lysine acetyltransferase that is involved in histone acetylation, thereby regulating gene expression and chromatin remodeling. Our results identified KAT5 as a central hub in both protein–protein interaction networks. KAT5, a member of the MYST protein family, contains a conserved domain with atypical zinc finger and histone acetyltransferase (HAT) domains, as well as a chromodomain. KAT5 has been implicated in various cellular activities, including DNA repair, apoptosis, transcriptional regulation, and cell proliferation [52,53], which are cellular activities of the cellular stress response and are likely to be directly related to the NF-kB pathway. Strangely, CS extract supplementation in PBMCs infected with PRRSV showed highly interconnected hubs of the network more than in the PRRSV group (Figure 4A,B). This may be because the CS extract is involved in establishing a connection with the surrounding genes related to the expression of the *KAT5* gene, which is an interesting point to explore in future studies.

This in vitro study reveals a unique transcriptomic signature for PRRSV infection with CS extract supplementation. Our genome-wide analysis highlights the activation of cellular stress response genes and other ubiquitously expressed genes. The complex biological processes involved in CS extract-mediated inhibition of viral replication in PBMCs underscore its potential role in antiviral immunity.

In conclusion, this study identified 274 DEGs between the PRRSV+CS and CTRL groups. In silico analysis suggested that *ZNF646* and *KAT5* may be potential candidate genes involved in the interaction between PRRSV-infected PBMCs and CS extract supplementation. Our findings suggest that supplementing PBMC cells infected with PRRSV with CS extract may modulate the stress response. This modulation appears to involve the NF-κB signaling pathway, potentially creating an unfavorable environment for viral survival, replication, and potentially hindering PRRSV infection. This study contributes to a deeper understanding of the immunomodulatory role of CS in the pathogenesis of PRRSV. Notably, our findings suggest that CS extract has the potential to be a candidate for modulating PRRSV infection. However, future studies will probe deeper into the potential relationship between CS extract and the highly interconnected *SLX4* and *KAT5* genes within the network. Additionally, in vivo studies using animal models will provide in-depth knowledge beyond the findings presented herein.

## 4. Materials and Methods

### 4.1. Plant Extract and Virus Preparation

The heartwood of *Caesalpinia sappan* (CS) was procured from the Samoeng District of Chiang Mai Province, Thailand (18°46′05.6″ N, 98°39′39.5″ E). The heartwood was cut and dried in a hot-air oven at 60 °C for 48 h. Subsequently, it was ground to a fine powder and sieved through a 1 mm mesh. The fine powder was subjected to maceration in 95% ethanol (3 × 28 L) at room temperature for a duration of 72 h. The combined crude ethanolic extracts were filtered through Whatman No. 1 filter paper and then rotary-evaporated at 40 °C to completely remove the solvent. The CS extracts were maintained at a temperature of −20 °C until they were required for further processing. To prepare the stock CS solution, approximately 1 mg of CS extract was weighed and dissolved in 1 mL of DMSO to achieve a 4 mM stock solution. Subsequently, this stock solution was diluted with complete RPMI medium to obtain a final working concentration of 40 µM before use in the experiments (Section 4.3).

The MARC-145 monkey kidney epithelial cell line (ATCC CRL-12231) was procured from the American Type Culture Collection (ATCC), Virginia, USA. Cells were grown in Dulbecco’s modified Eagle’s medium (DMEM) supplemented with 10% fetal bovine serum (Gibco) and 1% penicillin/streptomycin and incubated at 37 °C in 5% CO_2_ in a humidified incubator. The cells used in this study were at passage 23. PRRSV (VR2332 North American genotype) was propagated in MARC-145 cells, and the virus was titrated using IPMA and then stored at −80 °C. The virus titer was determined and expressed as TCID_50_ according to the Reed–Muench method.

### 4.2. Sample Collection and Processing

Blood samples (10 mL each) were obtained from six four-week-old weaned piglets via jugular venipuncture. The blood was collected into EDTA-containing tubes, following the protocol outlined in a previous study [54]. Within one hour of blood collection, the blood samples were diluted 1:1 with sterile phosphate-buffered saline (PBS). The tubes were gently inverted and centrifuged at 800× *g* for 10 min at 4 °C using an Allegra X-15R centrifuge (Beckman Coulter, Brea, CA, USA). The plasma and packed red blood cells were discarded, leaving only the buffy coat. The buffy coat was transferred to a new tube and diluted with PBS to a final volume of 5 mL. The buffy coat layer, containing peripheral blood mononuclear (PBMC) cells, was carefully layered onto Lymphoprep^®^ density gradient medium (Axis-Shield, Rodeløkka, Norway) with a density of 1.077 g/mL. The mixture was then centrifuged at 800× *g* for 20 min at 4 °C without braking. The cell interface at the PBMC layer was carefully collected and washed twice with sterile PBS supplemented with 1% fetal bovine serum (FBS, Invitrogen, Thermo Fisher Scientific, Waltham, MA, USA). The samples were centrifuged at 700× *g* for 5 min at 4 °C. The PBMC pellets were resuspended in 3 mL of complete RPMI medium (RPMI-1640 medium supplemented with 10% FBS, 2 mM L-glutamine, 100 μM non-essential amino acids, 50 μM 2-β-mercaptoethanol, and 1% antimicrobial and antifungal reagent).

### 4.3. PRRSV Infection and CS Extract Supplementation

The PBMC cells from each animal, obtained as described in Section 4.2, were divided into three treatment groups and aliquoted (1 × 10^6^ cells/well) into a 24-well flat-bottom cell culture plate (SPL^®^ SPL Life Sciences, Gyeonggi-do, Korea). The cell culture plates were incubated overnight at 37 °C in a 5% CO_2_ incubator. On the following day, the media in all wells were replaced with the designated treatment media. The first treatment (CTRL) consisted of complete RPMI medium only. The second treatment (PRRSV) involved stimulation with PRRSV of 1 × 10^6^ TCID_50_. The third treatment (PRRSV+CS) consisted of PRRSV (1 × 10^6^ TCID_50_) supplemented with CS extract at 40 μM final concentration. All treated cells were incubated at 37 °C and 5% CO_2_ incubator for an additional 6 h. Subsequently, the cells were washed with sterile PBS and centrifuged, and the cell pellets were harvested into RNAlater™ Stabilization Solution (Thermo Fisher Scientific) for further processing.

### 4.4. Library Preparation, Sequencing, and RNA-Seq Primary Analysis

RNA was extracted, quality controlled, and subjected to library construction using the TruSeq Stranded mRNA Library Prep Kit, following the protocol outlined in the TruSeq Stranded mRNA Reference Guide # 1000000040498 v.00. The RNA integrity number (RIN) values obtained from our experiments ranged from 8.2 to 9.7. Sequencing was performed on an Illumina platform at Macrogen, Gangnam-gu, Seoul, Korea. Raw data were generated through base calling using integrated real-time analysis (RTA). The resulting BCL/cBCL (base call) binary files were converted to the FASTQ format using bcl2fastq without adapter trimming. The total number of raw reads (paired-end, 151 bp) obtained from the CTRL, PRRSV, and PRRSV+CS samples ranged from 47,625,700 to 53,021,018. The percentage of bases with a Phred quality score greater than 30 (Q30) was between 92.2% and 93.2%.

### 4.5. Bioinformatic Analysis and Visualization of RNA-Seq Data

FastQC (Version 0.12.0) was used for quality control of the high-throughput sequencing data (FASTQ files) from the CTRL, PRRSV, and PRRSV+CS samples. Trimmomatic (version 0.32) was employed to trim, crop, and remove adapters from the Illumina FASTQ data, facilitating downstream analysis. The cleaned data were aligned to the pig genome assembly (susScr11) using Spliced Alignment of Transcripts (HISAT2), a fast and sensitive alignment program for mapping RNA-seq reads [55].

The read counts of aligned reads (BAM files) to the pig genome (susScr11.ncbiRefSeq.gtf, accession no. GCF_000003025.6_Sscrofa11.1), were summarized using FeatureCounts [56]. The counts of mapped reads for genes, exons, promoters, gene bodies, genomic bins, and chromosomal locations were extracted from the GTF/GFF3 format annotation (susScr11.ncbiRefSeq), available from the UCSC genome browser. The count data files from the CTRL, PRRSV, and PRRSV+CS treatments were analyzed using the DESeq2 package (version 1.44.0) to identify differentially expressed genes (DEGs) [57]. Genes with a log fold change (logFC) greater than 2.0 and a false discovery rate (FDR) less than 0.05 (Benjamini–Hochberg adjusted) were considered upregulated. Genes with a logFC less than −2.0 and an FDR less than 0.05 were considered downregulated. The gene-level expression matrix data generated from DESeq2 were uploaded to iDEP 1.1, a user-friendly web application for in-depth analysis [58]. iDEP 1.1 was used to perform data exploration, differential expression analysis, pathway analysis, and visualization. Pairwise data analysis (CTRL vs. PRRSV, CTRL vs. PRRSV+CS) and visualization were performed using R version 4.4.0 (Puppy Cup) with packages. Boxplots of transformed data, principal component analysis (PCA), heatmaps, volcano plots, MA plots, and UpSet plots were generated by RStudio 2024.04.2 Build 764 (Chocolate Cosmos). Gene tracks for specific genes corresponding to each treatment (CTRL, PRRSV, and PRRSV+CS) were constructed using BAM and BAI files mapped to the genomic coordinates of the pig reference genome (susScr11) in Integrative Genomics Viewer (IGV) version 2.18 [59]. Most resulting *p*-values were adjusted using the Benjamini–Hochberg procedure [60] to control the false discovery rate (FDR).

### 4.6. Functional Annotation, Pathway Analysis, and GO Enrichment Analysis

PANTHER Classification System (Released 20240226) was used to conduct enrichment analysis on gene sets in *Sus scrofa* [61]. The biological process was selected as the Gene Ontology (GO) aspect. A list of genes was generated, and this gene list was then uploaded to the STRING database (version 12.0, https://string-db.org/, accessed on 31 August 2024) to predict protein–protein interactions (PPI) in pigs (*Sus scrofa*). Both direct (physical) and indirect (functional) interactions were considered. To ensure high-confidence interactions, only those with a score of ‘medium’ or better (score ≥ 0.400) were selected for further investigation [62].

### 4.7. RNA Extraction and Validation of Gene Expression Using Real-Time PCR (qPCR)

Portions of the cryopreserved PBMC pellets in RNAlater, as detailed in Section 4.3, were subjected to RNA extraction with RNAzol^®^RT reagent (Sigma-Aldrich, St. Louis, MO, USA), employing minor modifications from the manufacturer’s protocol. Subse-quently, 5 μg of the extracted RNA was utilized for reverse transcription with iScript Reverse Transcription Supermix for RT-qPCR (Bio-Rad, Hercules, CA, USA), following the manufacturer’s guidelines. To validate the accuracy of the RNA-seq data, cDNA samples (100 ng) were subjected to quantitative PCR analysis to investigate gene expression. Six genes were arbitrarily chosen for confirmation: *AKAP3*, *ARHGA*, *HAUS3*, *PRRS58*, *PHOX2A*, and *NTS3B*. *GAPDH* served as the housekeeping gene. The SensiFAST SYBR Hi-ROX Kit (Bioline, Meridian Life Science, Memphis, TN, USA) was employed for gene expression quantification using the CFX96 Touch Real-Time PCR Detection System (Bio-Rad), with subsequent data analysis using CFX Maestro Software version 2.3. The qPCR protocol involved an initial polymerase activation step at 95 °C for 2 min, followed by 40 cycles of denaturation at 95 °C for 5 s and annealing/extension at 60 °C for 10 s. Data acquisition was performed at the end of the annealing/extension step. All reactions were performed in triplicate in 96-well reaction plates (20 μL per reaction). Primer sequences are provided in Table S3.

### 4.8. Statistical Analysis

The Student’s *t*-test was conducted to analyze mRNA expression levels (RT-qPCR) between the three groups (PRRSV versus CTRL and PRRSV+CS versus CTRL) using GraphPad Prism version 10.0 software (GraphPad, San Diego, CA, USA). A *p*-value < 0.05 was considered to be statistically significant. The results are the mean plus standard error mean (mean ± SEM).

### 4.9. Data Availability

The RNA-seq data in this study have been deposited at the NCBI Gene Expression Omnibus (GEO) under the accession number GSE277761.

## Data Availability

The data presented in this study are available on request from the corresponding author.

## Supplementary Materials

The following supporting information can be downloaded at: https://www.mdpi.com/article/10.3390/ijms252212285/s1.
