# Supplementary material for: Transcriptome Analysis of Porcine Immune Cells Stimulated by Porcine Reproductive and Respiratory Syndrome Virus (PRRSV) and Caesalpinia sappan Extract
Source: Int J Mol Sci. 2024 Nov 15;25(22):12285. doi: 10.3390/ijms252212285 (PMC11595159; doi:10.3390/ijms252212285)
Supplement: Supplementary file 1 [file ijms-25-12285-s001.zip › Table S3 Revision.pdf]

Supplementary Table 3. Primers and their sequences used in qRT-PCR analysis.

Gene	Accession	Direction	Sequence	Annealing Temp. (°C)	Size (bp)
AKAP3	NM_001195324.1	Forward	TTTGTCTCGGCCGTGAAAAG	60	142
		Reverse	ATTTGTCCTTGGCTCTGCTG		
ARHGAP9	XM_013997657.2	Forward	TTTGGCTGCCAGTTGGAATC	60	166
		Reverse	TGTCCACCAAGAAACGAAGC		
HAUS3	XM_021100924.1	Forward	GGAGCAGCATTGGATGAAGTTC	60	160
		Reverse	CCATCACCTGGCATTTGTTACG		
LARGE2	XM_021083119.1	Forward	AGACGCTCTTTCACACATGG	60	107
		Reverse	GCCAGAATAGTGCTTGTTAGGG		
KLHL40	XM_013981524.2	Forward	ATGATGGCCGCATTTTCGTG	60	173
		Reverse	AAAGCCACCGACAGCATAGAG		
NT5C3B	XM_021066994.1	Forward	TGTTCCACCCCAACATTCAC	60	114
		Reverse	ACACGGAGCTGTTCTTGTTG		
PHOX2A	XM_021062473.1	Forward	CCAGAACGTACACTGCAAAACC	60	106
		Reverse	TTGGATGCAAACAGGCGTTG		
PRSS58	XM_021079191.1	Forward	TTCAATGCAGCTGTGAGCAC	60	103
		Reverse	TGCAAGCTGGGAAATGTCTC		
GAPDH	AF017079.1	Forward	ACTCACTCTTCTACCTTTGATGCT	60	100
		Reverse	TGTTGCTGTAGCCAAATTCA		
